# DetectiV: visualization, normalization and significance testing for pathogen-detection microarray data

**DOI:** 10.1186/gb-2007-8-9-r190

**Published:** 2007-09-14

**Authors:** Michael Watson, Juliet Dukes, Abu-Bakr Abu-Median, Donald P King, Paul Britton

**Affiliations:** 1 Institute for Animal Health, Compton, Newbury, Berks RG20 7NN, UK; 2 Institute for Animal Health, Pirbright, Surrey GU24 0NF, UK

## Abstract

DNA microarrays offer the possibility of testing for the presence of thousands of micro-organisms in a single experiment. However, there is a lack of reliable bioinformatics tools for the analysis of such data. We have developed DetectiV, a package for the statistical software R. DetectiV offers powerful yet simple visualization, normalization and significance testing tools. We show that DetectiV performs better than previously published software on a large, publicly available dataset.

## Abstract 2

DetectiV is a tool for analyzing pathogen-detection microarray datasets that allows simple visualisation, normalisation and significance testing.

## Rationale

One of the key applications of metagenomics is the identification and quantification of species within a clinical or environmental sample. Microarrays are particularly attractive for the recognition of pathogens in clinical material since current diagnostic assays are typically restricted to the detection of single targets by real-time PCR or immunological assays. Furthermore, molecular characterization and phylogenetic analysis of these signatures can require downstream sequencing of genomic regions. Many microarrays have already been produced with the aim of characterizing the spectrum of micro-organisms present in a sample, including detection of known viruses [1-5], assessment of bioterrorism [6,7] and monitoring food quality [8].

However, the use of DNA microarrays for routine applications produces many challenges for bioinformatics. Firstly, probe selection is a difficult and time consuming process. There are a huge number of diverse species in nature, of which we have sequence information for only a tiny fraction. This makes it difficult to find oligonucleotides, either alone or in combination, that uniquely identify species of interest. Oligos may have homology to multiple species, which results in a complex and noisy hybridization pattern. Secondly, each nucleic acid sample tested will typically contain a mixture of DNA and RNA from the organism of interest, the host and from a variety of contaminants, which may all contribute to the resulting microarray profile. Furthermore, this may be complicated by the presence of multiple, possibly related, pathogen species, making it difficult to separate patterns due to cross-hybridization from a true positive result.

Urisman *et al*. [9] have previously reported E-Predict, a computational strategy for species identification based on observed microarray hybridization patterns. E-Predict uses a matrix of theoretical hybridization energy profiles calculated by BLAST-ing completely sequenced viral genomes against the oligos on their array, and calculating a free energy of hybridization. Observed hybridization profiles are then compared to the theoretical profiles using a similarity metric, and a *p *value calculated using a set of experimentally obtained null probability distributions. E-Predict has been shown to produce useful results in a number of situations. However, at present, E-Predict does not contain any tools for visualization, and requires extensive customization and calculation before it is applicable to new arrays. Also, E-Predict is only available as a CGI script for Unix/Linux platforms.

We present DetectiV, a package for R [10] containing functions for visualization, normalization and significance testing of pathogen detection microarray data. R is a freely available statistical software package available for Windows, Unix/Linux and MacOS, meaning DetectiV is a platform independent solution. DetectiV uses simple and established methods for visualization, normalization and significance testing. When applied to a publicly available microarray dataset, DetectiV produces the correct result in 55 out of 56 arrays tested, an improvement on previously published methods. When applied to a second dataset, DetectiV produces the correct result in 12 out of 12 arrays.

## Implementation

DetectiV is implemented as a package for R, a powerful, open-source software package for statistical programming [10]. Many packages for R already exist for the analysis of biological datasets, including microarray data, and the bioconductor project [11] is just one example of a group of such packages. As it is implemented in R, DetectiV easily integrates with many of the packages available for microarray analysis, including limma [12], marray [11] and affy [13].

DetectiV is written in the native R language and uses standard functions within R. As R is available on Microsoft Windows, Unix (including linux) and MacOS, DetectiV represents a platform independent solution for the analysis of pathogen-detection microarray data.

**Figure 1:**
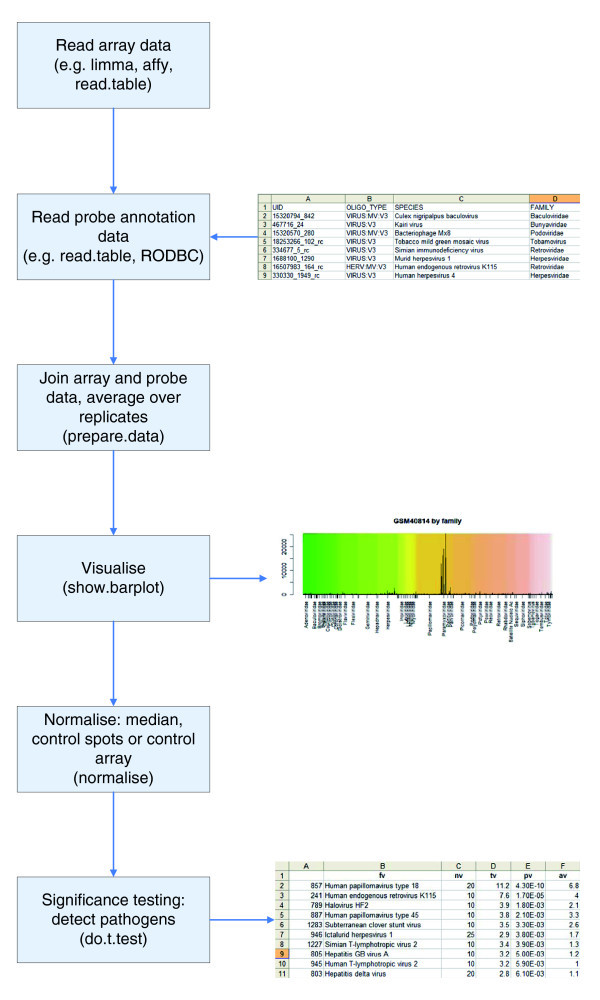
Flow of information, and steps taken, when analyzing pathogen detection microarray data using DetectiV.

The flow of information through DetectiV is shown in Figure 1. The basic dataset required is a matrix of data, with rows representing probes on the array, and columns representing measurements from individual microarrays. This dataset is easily produced from data structures created by limma [12], which includes functions for reading in many common microarray scanner output formats, and affy [13], which provides functions for reading in affymetrix data. Commonly, researchers will have an additional file of information giving details about each probe. In the case of pathogen detection arrays, this file will most often contain the type, species, genus and other classification data for the pathogen to which each probe is designed. It should be noted that there may be more than one entry in this file for each probe; for example, if a given probe is thought to hybridize to multiple pathogens. In text format, these may be read in using the native read.table command, or in excel format using the RODBC library.

Once these two datasets are in R, DetectiV prepares them for analysis using the prepare.data function. This function joins the array data to the probe information data based on a unique ID. The researcher may choose to subtract local background if appropriate. The default at this stage is to average over replicate probes, again based on a unique ID. This will result in a single value for each unique probe for each array. The data will have one or more columns of extra information from the annotation file, and these columns will be used to group the data for further analysis.

**Figure 2:**
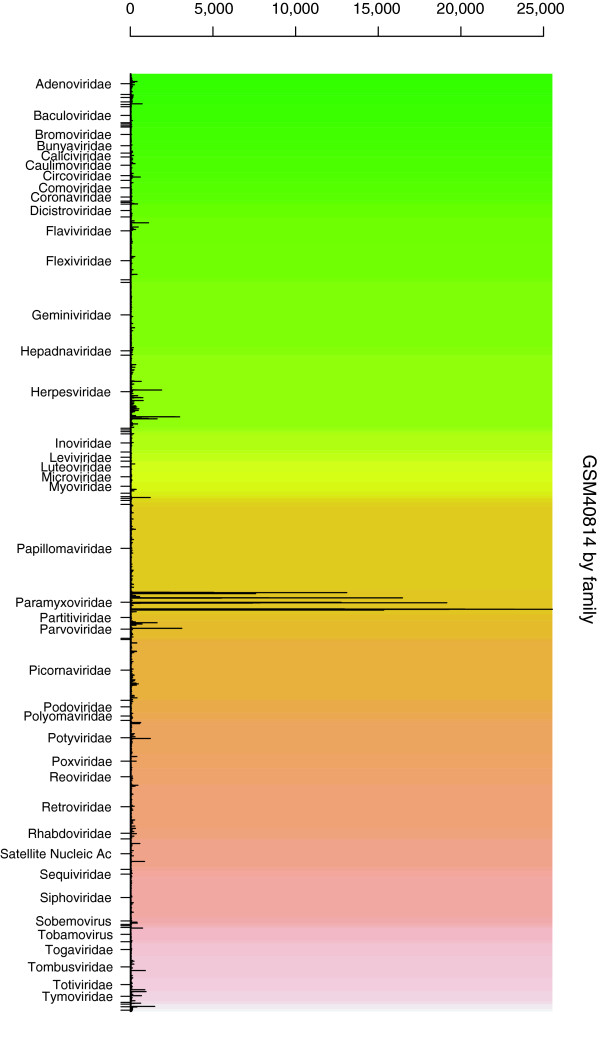
GSM40814 by family. Example barplot from DetectiV showing data from a virus detection microarray. The sample included amplified RNA from nasal lavage, positive for respiratory syncytial virus by DFA. Oligos have been averaged over replicates and grouped according to virus family. Each unique oligo is represented by a single bar. Each virus family has a unique background color. The y-axis is raw intensity.

Researchers will wish to visualize their data in order to compare the hybridization signals for the probes recognizing the different pathogen signatures. DetectiV provides a function called show.barplot for this. The output from prepare.data is passed to the function, along with the name of the column containing the variable by which the data will be grouped, referred to here as *group*. An example in pathogen detection data may be species, genus, family, and so on. The data are sorted into unique groups as defined by the unique values of *group*. A barplot is drawn, with one bar per unique probe. Probes from the same group are drawn together. Each group is represented by a unique background color, enabling the user to easily visualize the different groups. An example output is shown in Figure 2. This sample comes from Urisman *et al *[9] and represents data from a virus detection microarray hybridized with amplified RNA from nasal lavage, positive for respiratory syncytial virus by direct fluorescent antibody (DFA) test. The *group *chosen here is virus family. It is quite clear from this image that there is a virus from the family *Paramyxoviridae *present in the sample, demonstrated by the high bars associated with that family.

**Figure 3:**
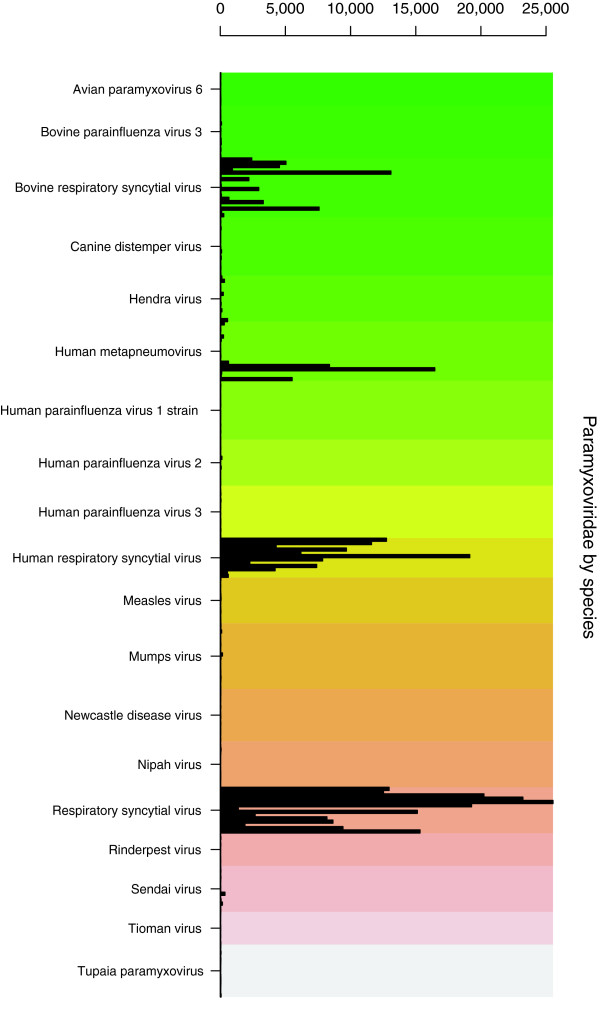
GSM40814 Paramyxoviridae by species. Example barplot from DetectiV showing data from a virus detection microarray. The sample included amplified RNA from nasal lavage, positive for respiratory syncytial virus by DFA. Only oligos representing species from the Paramyxoviridae family are shown. Oligos have been averaged over replicates and grouped according to virus species. Each unique oligo is represented by a single bar. Each virus species has a unique background color. The y-axis is raw intensity.

These images are often very large, and so DetectiV offers the ability to subset the data before plotting by using the get.subset function. Figure 3 shows a similar barplot using a subset of the data: only those oligos representing species that belong to the *Paramyxoviridae *family. It is clear from this image that those oligos representing different groups/species of respiratory syncytial virus have the highest intensity, as we would expect, although there is cross-hybridization with oligos for human metapneumovirus (another paramyxovirus in the same sub-family: Pneumovirinae).

DetectiV may also carry out normalization and significance testing. For this, there is the function normalise. Here, the aim of normalization is to represent the data in relation to a negative control. The idea is that if the values for each probe are divided by the negative control and then the log2 taken, then the data should be normally distributed, and each group should have a mean of zero (providing a pathogen is not present). Traditional statistical tests can then be used to test if any group of probes is significantly different from zero. DetectiV offers three methods of normalization, each using a different 'type' of negative control, and these are summarized in Table 1.

**Table 1:** DetectiV normalization methods

Method	Normalized statistic	Terms
Median	$\log_{2}\left(x_{j}^{i}/{\tilde{x}}_{j}\right)$	Where $x_{j}^{i}$ is the value for probe *i *on array *j *and ${\tilde{x}}_{j}$ is the median value for all probes on array *j*
Control	$\log_{2}\left(x_{j}^{i}/{\bar{c}}_{j}\right)$	Where $x_{j}^{i}$ is the value for probe *i *on array *j *and ${\bar{c}}_{j}$ is the mean value for control oligo *c *on array *j*
Array	$\log_{2}\left(x_{j}^{i}/x_{c}^{i}\right)$	Where $x_{j}^{i}$ is the value for probe *i *on array *j *and $x_{c}^{i}$ is the value for probe *i *on control array/channel *c*

Explanation of the three normalized statistics offered by DetectiV.

The median method calculates the global median value for each array. It should be noted that this method assumes that most probes will not hybridize to anything. If this assumption is false then this method should not be used. However, if the assumption holds, then the median is a good representation of that value we would expect to see from probes that have not hybridized to anything.

The control method relies on specific negative controls having been spotted on the array. The researcher may then choose one of these controls, and the mean value is calculated for that control for each of the arrays. The mean control value for each array is then used as a divisor for each probe on their respective arrays.

Finally, the array method utilizes an entire control array or channel. In this instance, an entire array is chosen to be the negative control, and all probe values are divided by their respective elements from the control array. An obvious example for a control array may be RNA from a known uninfected animal. The control array therefore has a value for each specific probe representing that value we would expect to see if that specific probe has not hybridized to anything.

In all instances, after taking the log2, groups of probes that have not hybridized to anything should be normally distributed and have mean zero. We can therefore split the probes into groups and perform a *t*-test for each one. DetectiV does this using the do.t.test function. The normalized (or raw) data are split into groups as defined by the unique values of a user defined annotation column. Providing each group has more than two probes, a *t*-test is performed to test the difference of the observations from zero. The average value is also calculated. The output is a table, sorted by *p *value.

## Methods and data analysis

The data used were downloaded from the Gene Expression Omnibus (GEO) [14], accession number GSE2228. The array platform for this data is GEO accession GPL1834, and includes over 11,000 oligos representing over 1,000 viral and bacterial species [4].

The dataset itself consists of 56 arrays including 15 independent HeLa RNA hybridizations, 10 independent nasal lavage samples positive for respiratory syncytial virus, 7 independent nasal lavage samples positive for influenza A virus, a serum sample positive for hepatitis B virus, a nasal lavage sample positive for both influenza A virus and respiratory syncytial virus, and culture samples of 11 distinct human rhinovirus serotypes.

Both DetectiV and E-Predict [9] have been used to analyze the data. For DetectiV, the data were not corrected for local background. Missing, negative and zero values were set to a nominal value of 0.5. Intensities were averaged across replicate probes. Median normalization was then carried out, followed by a *t*-test grouping the data by virus species. Probes representing actin, GAPDH and Line_Sine were filtered from the results. Results were first filtered such that groups had a normalized log2 ratio greater than or equal to 1 (a ratio of two to the control) and then sorted by *p *value. This method will be referred to as DetectiV.

For E-Predict, default values for all parameters were used, and are shown in Table 2. Data points were corrected for local background, as per the examples in Urisman *et al*. [9]. E-Predict filters out 266 oligos by default, and this setting was kept. In all cases, E-Predict carried out two iterations, although only results from the first iteration are shown here. The best performing method of interpreting the results was to take those species with a *p *value ≤ 0.05 and sort by distance (termed E-Predict.dist). Note that this is the method cited in [9], example 3, used to demonstrate E-Predict's ability to detect SARS.

**Table 2:** E-Predict parameters

Parameter	Value
user_wts	MV_72worst_medRaw500_badYdens
norm_opt	Sum
energy_filter	undef
ematrix	22/07/2004
ematrix_norm	Quadratic
ematrix_efilter	30
dist_metric	Pearson Uncentered
iterate	2
top_oligos	5
top_genomes	5
top_fams	5
sort_by	Distance|P value
eclust	None

Parameters used for input into E-Predict.

Pathogen detection arrays have also been implicated in the discovery of SARS. Urisman *et al*. [9] reported that although their original platform did not contain oligos designed to SARS, once the SARS genome had been published, it was possible to recalculate the energy matrix for E-Predict and find that the energy profile for SARS was the top hit (after taking those viruses with low *p *values and sorting by distance). We have applied DetectiV to the same dataset (GEO accession GSE546). To include oligos for SARS, we searched a database of oligo sequences on the array with sequence NC_004718 from RefSeq using NCBI blast. There were 61 oligos on the array that hit the SARS genome with greater than 80% identity across an alignment of 20 bp or more. In the analysis, these oligos were assigned as representative of two viruses: their original virus and SARS. The data were median normalized and a *t*-test carried out using DetectiV.

Finally, having established that DetectiV compares favorably with previously published software, we have validated the DetectiV software by applying it to a second dataset. The data used were downloaded from the GEO [14], accession number GSE8746. The array platform for this data is GEO accession GPL5725, and consists of 5,824 oligos representing over 100 viral families, species and subtypes. The dataset itself consists of 12 arrays, 4 hybridized with RNA from cell cultured foot-and-mouth disease virus (FMDV) type O, 3 hybridized with RNA from FMDV type A, 1 hybridized with RNA from a sheep infected with FMDV type O, and 4 hybridized with cell-cultured avian infectious bronchitis virus (IBV). Analysis using DetectiV was carried out as described above.

## Results and comparison

We present here results from two methods of analysis, termed DetectiV and E-Predict.dist, as described above. There are 56 arrays in the dataset, the expected results of which are known. Each array was hybridized with RNA containing a single virus, except GSM40845, which was infected with both influenza A and respiratory syncytial virus. We assigned a correct result for each method if the top hit from the analysis was the same as the known infectious agent or, if that agent was not represented on the array, the top hit was a very closely related virus. In the case of GSM40845, we report a correct result if both viruses were at the top of the reported hits, to the exclusion of other virus species (but not closely related strains).

Additional data file 1 gives the top hit for both analysis methods in all 56 arrays. As can be seen, DetectiV generated a correct result in 55 out of the 56 arrays. In comparison, the E-Predict.dist method gave a correct result in 53 out of the 56 arrays. These results are discussed in greater detail below.

### DetectiV

Full results for each of the arrays can be found on the DetectiV website [15]. Within the 55 correct results, there are three classes that require slightly different interpretation, examples of which are GSM40806, GSM40810 and GSM40817. Results for these arrays are given in Table 3.

**Table 3:** Typical results from DetectiV

GSM40806			GSM40810			GSM40820		
		
Virus	*p *value	Mean	Virus	*p *value	Mean	Virus	*p *value	Mean
Human papillomavirus type 18	4.1E-10	6.8	Human rhinovirus sp.	9.9E-12	4.1	Human herpesvirus 5	5.3E-16	0.57
Human endogenous retrovirus K115	0.000016	4	Human rhinovirus A	2.3E-09	4.1	Respiratory syncytial virus	1.1E-09	4.26
Halovirus HF2	0.0017	2.1	Enterobacteria phage M13	2.2E-07	5.7	Human rhinovirus sp.	5.9E-08	0.75
Human papillomavirus type 45	0.002	3.3	Human rhinovirus 16	6.2E-07	3.5	Human rhinovirus B	1.4E-07	0.47
Subterranean clover stunt virus	0.0032	2.6	Human rhinovirus 1B	0.000001	3.5	Human rhinovirus A	6E-07	0.75

Top five hits from three microarrays showing typical results from DetectiV. All have been sorted by *p *value. GSM40806 and GSM40810 have been filtered such that mean ≥ 1.

Array GSM40806 was hybridized with amplified HeLa RNA, and the top hit from DetectiV is human papillomavirus type 18, as expected. This virus has both the smallest *p *value and largest mean normalized log ratio. There is also clear distinction between the top hit and the rest of the hits below; there are orders of magnitude between the values for both the *p *value and the mean normalized log ratio. The other hits in the table are expected as a result of hybridization by the virus and host RNA to non-specific probes on the array. However, the clear distinction in both the *p *value and mean log ratio identify human papillomavirus type 18 as the top, and only, hit.

GSM40810 was hybridized with RNA containing human rhinovirus 28. There are 24 distinct groups of human rhinoviruses represented on the array, including a group of oligos for all members ('human rhinovirus sp.), one each for human rhinovirus A and B, and several groups for distinct serotypes. Human rhinovirus 28 is not one of those serotypes specifically targeted by the array; however, as a serotype of the human rhinovirus A species, we would expect the groups for human rhinovirus sp. and human rhinovirus A to be prevalent amongst the results. As can be seen from Table 3, the top hit from DetectiV is human rhinovirus sp., closely followed by human rhinovirus A, the expected result. The reason we have highlighted this array, however, is that the result for Enterobacteria phage M13 shows a higher mean normalized intensity than any of the rhinovirus groups. This is representative of a class of result from DetectiV whereby a virus group has a higher mean normalized log ratio, but a larger *p *value, than the top hit. Here, as in GSM40806, we see orders of magnitude between the *p *value for the top hit and that for Enterobacteria phage M13, which identifies human rhinovirus as being the infectious agent, but in this case we cannot rely on the mean normalized intensity. In this particular instance, Enterobacteria phage M13 is represented by 10 oligos, all of which have intensities far greater than the global median, but which vary considerably between 982 and 18,864. These high values may be due to hybridization with a cloning vector.

Finally, array GSM40817 was hybridized with respiratory syncytial virus. The results are again shown in Table 3, but for this array only, they have not been filtered on mean normalized intensity. Human herpesvirus 5 has by far the smallest *p *value of any of the virus groups; however, it also has a very small mean normalized log ratio. The correct hit, respiratory syncytial virus, has the second smallest *p *value, but has a much larger mean normalized log ratio. This represents the final class of result seen by DetectiV, where the correct virus group does not have the smallest *p *value, but does have a much larger mean normalized log ratio than those groups that have smaller *p *values. The small *p *value of respiratory syncytial virus combined with the large mean normalized log ratio identifies respiratory syncytial virus as the only infectious agent. In this instance, human herpesvirus 5 is represented by 241 oligos, 167 of which are greater than the global median, but all of which have intensities less than 1,000. This could be due to the oligos for human herpesvirus 5 having distant homology with the infectious agent or host cell.

These three types of result are typical of DetectiV, and explain why both the *p *value and the mean normalized log ratio must be taken into account when interpreting the results. Thus, if the results from DetectiV are filtered such that only viruses whose mean normalized log ratio is ≥ 1, and then sorted by *p *value, the three scenarios described here are accounted for, and we obtain the correct result in 55 out of the 56 arrays.

The single incorrect result for DetectiV comes from GSM40816, which reports human herpesvirus 7 as the top hit, whereas the infectious agent was in fact respiratory syncytial virus. The top five hits for this array using the DetectiV method are shown in Table 4. As can be seen, bovine respiratory syncytial virus and respiratory syncytial virus are second and third, respectively. Both respiratory syncytial virus and bovine respiratory syncytial virus have higher mean values than human herpesvirus 7, although the latter has a smaller *p *value and a mean value that is above the cut-off of 1. Had the results been filtered for *p *value ≤ 0.5 and then ordered by average value, then the top hit would have been respiratory syncytial virus; similarly, if a cut-off of 2 had been applied instead of 1, a correct result would have been reported. However, across the entire dataset these methods of interpreting the results perform worse than the DetectiV method described above. It is worth noting here that for this array, E-Predict gives the correct top hit.

**Table 4:** Incorrect DetectiV result

Virus	*p *value	Mean
Human herpesvirus 7	8.60E-06	1.7
Bovine respiratory syncytial virus	2.70E-04	2
Respiratory syncytial virus	3.30E-04	3.2
Ictalurid herpesvirus 1	1.50E-03	1.7
Human herpesvirus 6B	1.50E-03	1.8

Top five hits from the DetectiV method from array GSM40816. The sample for this array was found to contain respiratory syncytial virus by DFA.

### E-Predict

The results from E-Predict follow similar patterns to those of DetectiV. In most cases it is obvious which virus is the infectious agent, either by examining the *p *value, the similarity or both together. Full results can be seen on the DetectiV website [15]. However, there are certain results reported by E-Predict where it is impossible to obtain the correct result no matter which combination of *p *value and similarity is used. These arrays are arrays are GSM40809, GSM40821 and GSM40847, and the top five results for these arrays can be seen in Table 5.

**Table 5:** Incorrect E-Predict results

GSM40809			GSM40821			GSM40847		
		
Virus	*p *value	Similarity	Virus	*p *value	Similarity	Virus	*p *value	Similarity
Human enterovirus D	0.000043	0.258894	Orangutan hepadnavirus	0.002291	0.148865	Human enterovirus B	0.000014	0.386095
Human rhinovirus B	0.000045	0.267815	Hepatitis B virus	0.002376	0.147182	Human enterovirus A	0.000016	0.378912
Human enterovirus C	0.000052	0.254504	Woodchuck hepatitis B virus	0.002716	0.10964	Human echovirus 1	0.000022	0.414618
Enterovirus Yanbian 96-83csf	0.000094	0.276873	Woolly monkey hepatitis B Virus	0.00284	0.128919	Enterovirus Yanbian 96-83csf	0.000022	0.412299
Human echovirus 1	0.000134	0.253816	Arctic ground squirrel hepatitis B virus	0.003227	0.103357	Human enterovirus D	0.000026	0.296065

Top five results from the E-Predict.dist method for arrays GSM40809, GSM40821 and GSM40847. In all cases results are ordered by *p *value.

GSM40809 was hybridized with RNA containing human rhinovirus 26. Again, this is a serotype not specifically targeted by the array; however, as a serotype of human rhinovirus B we would expect the 'human rhinovirus sp.' and 'human rhinovirus B' groups to be the top hits (this is the case for DetectiV). However, E-Predict reports human enterovirus D as having the smallest *p *value, and enterovirus Yanbian 96-83csf as having the largest similarity. The top five hits reported in Table 5 for this array all have similar *p *values and similarity measures, and there is no way of sorting or filtering the results such that human rhinovirus B becomes the top hit. Without the *a priori *knowledge that human rhinovirus 26 was the infectious agent, it would be more likely to conclude that a species of enterovirus was present in the sample. It is no surprise that these viruses are being confused, as they are related viruses from the Picornaviridae family. However, DetectiV is capable of calling the correct result in this instance, whereas E-Predict is not.

Array GSM40821 was infected with hepatitis B virus but E-Predict reports orangutan hednavirus as having both a smaller *p *value and a higher similarity. This is not that surprising given that hepatitis B and orangutan hepadnavirus are closely related; however, the fact remains that with no *a priori *knowledge, the only logical conclusion from this result would be that the infectious agent was orangutan hepadnavirus. Again, DetectiV calls this array correctly.

Finally, array GSM40847 was hybridized with RNA containing human rhinovirus 87. Again, this is a serotype not specifically targeted by the array, and is not present in the NCBI taxonomy database [16] at the time of writing. We can therefore expect the 'human rhinovirus sp.' group to be high amongst the results (in fact, it is the top result for DetectiV). E-Predict reports human enterovirus B as having the smallest *p *value and human echovirus 1 as having the largest similarity. In fact, E-Predict does not report any rhinovirus oligos in the first iteration at all, and it is only in the second iteration that the group human rhinovirus A is reported as significant.

In the three cases outlined above, there is no clear way of distinguishing the incorrect virus from the correct one. There is also no consistent method of sorting or filtering the results that would give the correct results. In these three cases, E-Predict is unable to distinguish closely related virus species and serotypes. We have reported here the best performing method of interpreting E-Predict results, whereby virus groups with a *p *value ≤ 0.05 are sorted by distance. This results in a success rate of 53 out of 56 arrays.

### DetectiV and SARS

The top five hits from the analysis of the SARS dataset can be found in Table 6. As can be seen, the top hit is SARS, with the lowest *p *value and the highest mean normalized log ratio. SARS is distinct from the other viruses, having a *p *value three orders of magnitude lower than the second top hit.

**Table 6:** DetectiV results for SARS array

Virus	*p *value	Mean
SARS	8.43E-09	1.906095
Human herpesvirus 7	3.29E-06	1.292008
Simian retrovirus 2	4.27E-05	1.328653
Coliphage alpha3	6.08E-05	1.113462
Transmissible gastroenteritis virus	7.88E-05	1.463675

Top five results from the DetectiV method of analyzing array GSM8528 from GEO accession GSE546. The sample hybridized to the array contained the SARS virus.

### Validation

Full results can be found on the DetectiV website [17]. The top hit from DetectiV for each of the 12 arrays from GSE8746 can be found in Table 7. As can be seen, DetectiV clearly identifies the infectious agent in all 12 cases. DetectiV works for both the cell-cultured samples and the infected sheep, and shows the ability of the array to distinguish between different subtypes of FMDV.

**Table 7:** Top hit for GSE8746

Array	RNA	Top hit	*p *value	Mean
GSM216542	Amplified RNA from cell cultured FMDV type O	FMDO	1.51E-25	2.296645
GSM217164	Amplified RNA from cell cultured FMDV type O	FMDO	1.07E-45	3.513068
GSM217167	Amplified RNA from cell cultured FMDV type O	FMDO	2.36E-48	3.446262
GSM217169	Amplified RNA from cell cultured FMDV type O	FMDO	5.91E-30	2.827877
GSM217172	Amplified RNA from cell cultured FMDV type A	FMDA	6.96E-30	3.560941
GSM217175	Amplified RNA from cell cultured FMDV type A	FMDA	8.71E-14	1.553392
GSM217177	Amplified RNA from sheep infected with FMDV type O	FMDO	1.12E-27	2.431874
GSM217180	Amplified RNA from cell cultured FMDV type A	FMDA	2.97E-33	3.609092
GSM217183	Amplified RNA from cell cultured Avian IBV	IBV	1.05E-21	5.262134
GSM217184	Amplified RNA from cell cultured Avian IBV	IBV	3.49E-33	7.958662
GSM217186	Amplified RNA from cell cultured Avian IBV	IBV	6.20E-33	7.827526
GSM217188	Amplified RNA from cell cultured Avian IBV	IBV	1.44E-35	8.0118

The top hit from DetectiV for the 12 arrays from the GSE8746 dataset. DetectiV produces the correct result in all 12 cases.

## Discussion

Developing a quick and reliable test for the presence/absence of thousands of bacterial and viral species in a single experiment is an attractive proposition, and a function that DNA microarrays are ideally suited to. Microarrays are extremely high-throughput and relatively cheap. In the case of pathogen detection, the aim must be to quickly and clearly identify those pathogens present in a sample with high confidence, keeping false positives and false negatives to a minimum.

However, the data from such microarrays pose many problems. Firstly, oligos may not be unique to the species they are designed to. For certain species it is impossible to find a large number of oligos that are unique only to that virus that meet the criteria for oligo selection. This is particularly problematic for closely related species and strains. In such cases, the 'best' oligos are added to the array, in the knowledge that multiple viruses may hybridize to them. This leads to noisy signals across multiple virus families, species and serotypes. Secondly, infected biological samples may contain many different virus species and strains, making interpretation difficult. Thirdly, it is known that certain oligos simply do not work, even when the array is hybridized with the species that those oligos were designed to. Without testing the array with each virus, we are incapable at present of predicting which oligos will work and which will not. With thousands of species per array, many of which cannot be cultured *in vitro*, it is unfeasible to challenge arrays with every species. Finally, we of course do not know, nor can we ever know, the complete genome sequence of every virus we may encounter. Therefore, though we think we have oligos unique to a species or strain, that is only ever in the context of our knowledge at the time of design, and they may not in fact be unique.

Despite these problems, many species detection arrays have been developed [1-5]. However, reliable methods of data analysis have been rare. Initial methods included visual inspection of the array [4] and clustering [18], both of which are subjective and time-consuming. To combat this, Urisman *et al*. [9] have proposed a more robust method, E-Predict. E-Predict utilizes a pre-calculated energy matrix for each oligo on the array and uses a variety of normalization and similarity metrics to calculate a *p *value and similarity for each virus. The advantages of E-Predict are that it is quantitative, produces good results and is extensible, through the extension of the energy matrix. The disadvantages of the software are a lack of visualization tools, the need to customize parameters for different array platforms and hybridization conditions, and the availability of the software only as a CGI script on the Unix/Linux platform.

We have developed DetectiV, a package for R containing visualization, normalization and significance testing functions for pathogen detection data. DetectiV uses simple and well established visualization and statistical techniques to analyze data from pathogen detection microarrays. DetectiV offers a powerful visualization option in the form of a barplot, enabling researchers to quickly and easily identify possible infectious agents. Data can then normalized to a negative control (be that a specific probe, array or the global median), transformed by taking the log2 and then subjected to a *t*-test for each species on the array. Oligos are allowed to represent any number of viruses, and thus any analysis is easily extensible by simply updating the list of which oligos represent which species.

DetectiV requires minimal set up and configuration, requiring only an additional file detailing which species each oligo represents. In the majority of cases, these files will already exist. It is then possible to apply DetectiV 'out of the box' to any array data that is readable by R or bioconductor. DetectiV requires no training, configuration or customization specific to each array. DetectiV is available as a package for R on both Windows and Linux/Unix, and as such may be considered platform-independent.

In this study, DetectiV produced the correct result in 55 out of 56 arrays, by filtering for viruses with a mean normalized log ratio greater than 1 and then sorting by *p *value. We make the distinction here between biological and statistical significance. A statistically significant result may be obtained by a group of oligos that display intensities only marginally larger than the negative control (in this case the global median intensity). This is demonstrated by human herpesvirus 5 on array GSM40820 (Table 3). However, we know that from a biological perspective, we would expect to see intensities far higher than the negative control, and that intensities only marginally higher result from low homology between the probe and the sample. We can therefore use the statistical significance (*p *value) in combination with our idea of biological significance (the mean normalized log ratio) to successfully call the correct result in over 98% of the arrays.

In the majority of cases there is a clear difference in the *p *value, the mean normalized log ratio, or both, between the correct hit and subsequent hits, allowing for both automatic and manual detection of true and false positives. However, this does require careful interpretation. Both DetectiV and E-Predict predict multiple, significant matches on all of the arrays. When using DetectiV, it is only when looking for major changes between the top hit and subsequent hits, in terms of *p *value or mean log ratio, that it is possible to separate the true positives from the false positives. In many cases, using automatic rules will result in the correct result; however, there will inevitably be borderline cases where human inspection of the results is required. This is all the more important when considering the possible economic impacts of a false positive for certain species. At present, the safest way to employ such arrays, and their analysis methods, may be simply as a first step towards identifying infectious agents, informing researchers about which viruses they should test for using more conventional methods.

The results from the application of DetectiV to the SARS dataset are encouraging. Here, oligos designed to SARS were not present on the array. However, using a simple NCBI blast search, it was possible to extend the range of viruses covered by the array to include SARS - 61 existing oligos showing significant homology to the SARS genome. On application of DetectiV to the updated data, SARS was the top hit. Not only does this offer the promise of being able to extend the coverage of the array without adding further oligos, it also suggests that it is possible to detect viruses without having any unique oligos. This may inform the oligo selection process - it may be equally desirable to have multiple, non-unique oligos to represent a species as it is to have a few that are unique.

The results from the application of DetectiV to a second dataset are also encouraging, with the correct result being the top hit in all 12 cases. Of particular interest is the ability of the array, and DetectiV, to distinguish not only between separate viral species, but also between different subtypes of FMDV. It should be noted that in order to apply DetectiV to a second dataset from a completely different array to the first dataset, the user only has to change the GEO accession number and the number of arrays within that dataset. This compares favorably with E-Predict, which would require a separate training dataset from the second array, the calculation of a large and complex sequence similarity matrix and the optimization of several parameters.

There are a number of ways in which DetectiV may be developed. In terms of visualization, better browsing capabilities of the barplots would be desirable, perhaps using a web-interface. In terms of the analysis, we may borrow ideas from gene expression arrays. For example, limma uses an empirical Bayes method to shrink each gene's standard error towards a common value, and has been shown to perform better than standard statistical methods [12]. It may be that we can apply a similar method here to shrink the standard error for each virus species towards a common value, thus increasing sensitivity. It may also be possible to apply multiple-testing procedures to the resulting *p *values. The Bonferroni correction may be appropriate, in which the *p *values are multiplied by the number of comparisons, or a more conservative approach may be needed, such as that suggested by Benjamini and Hochberg [19], in order to control the false discovery rate.

In conclusion, DetectiV is a highly accurate tool for the analysis of pathogen detection microarray data, offering simple but powerful visualization, normalization and significance testing functions. DetectiV performs better than previously published software on a publicly available microarray dataset. DetectiV is available as a package for R, a platform-independent statistical software package, and requires little configuration or customization. It is released under the GNU General Public License and may be downloaded from the DetectiV website [20].

## Abbreviations

DFA, direct fluorescent antibody; FMDV, foot-and-mouth disease virus; GEO, Gene Expression Omnibus; IBV, infectious bronchitis virus.

## Authors' contributions

Michael Watson wrote and tested the DetectiV software. Juliet Dukes and Abu-Bakr Abu-Median designed the visualization styles in DetectiV, tested the software and produced the data in GSE8746. Donald King and Paul Britton tested the software and helped produce the data in GSE8746.

## Additional data files

The following additional data are available with the online version of this paper. Additional data file 1 is a table listing the top hit for all 56 arrays using both the Detectiv and E-Predict.dist methods. DetectiV produced a correct result in 55 out of 56 arrays, and E-Predict produced a correct result in 53 out of 56 arrays.

## Supplementary Material

Additional data file 1 DetectiV produced a correct result in 55 out of 56 arrays, and E-Predict produced a correct result in 53 out of 56 arrays. Click here for file
